# Limb-onset amyotrophic lateral sclerosis patients visiting orthopedist show a longer time-to-diagnosis since symptom onset

**DOI:** 10.1186/1471-2377-13-19

**Published:** 2013-02-09

**Authors:** Osamu Kano, Konosuke Iwamoto, Hirono Ito, Yuji Kawase, Derek Cridebring, Ken Ikeda, Yasuo Iwasaki

**Affiliations:** 1 Department of Neurology, Toho University Omori Medical Center, 6-11-1 Omorinishi, Ota-ku, Tokyo, 143-8541, Japan; 2 Department of Bioinformatics and Bioengineering, The Methodist Hospital Research Institute, 6670 Bertner Street, Houston, TX, 77030, USA

**Keywords:** Amyotrophic lateral sclerosis, Initial symptom, Bulbar onset, Limb onset, Neurologist, Orthopedist

## Abstract

**Background:**

There have been several reports concerning the survival time after symptom onset in patients with amyotrophic lateral sclerosis (ALS). However, little is known about how the choice of physician (i.e., general practitioner, neurologist, etc.) may affect the time it takes for a diagnosis of ALS to be made.

**Methods:**

We conducted a retrospective study, covering a 20-year period, comparing the type of physician first consulted by an ALS patient at the time of initial symptoms and the amount of time that elapsed to the final diagnosis of ALS. A total of 202 patients were diagnosed and stratified according to the onset of ALS symptoms (bulbar onset [BO] and limb onset [LO]). We noted the type of physician first seen by the patient. The diagnostic interval was calculated as the time between onset of symptoms and the final diagnosis of ALS.

**Results:**

A total of 202 ALS patients were examined. Clinical BO and LO was observed in 78 (36.6%) and in 124 (61.4%) of these patients, respectively. The type of physician examining these patients at the first symptoms of disease was as follows (BO and LO): neurologist (38.5% and 25.8%), general practitioner (14.1% and 35.5%), orthopedist (12.8% and 35.5%), otolaryngologist (15.4% and 0%), and neurosurgeon (14.1% and 3.2%). Mean diagnostic interval (standard deviation) for patients with either set of symptoms was 13.1 (6.5) months; the diagnostic interval of patients with BO and LO was 9.2 (4.5) and 15.2 (7.7) months, respectively. ALS diagnosis in LO patients was delayed by more than 10 months when the patient first consulted an orthopedist rather than a neurologist.

**Conclusion:**

More than 50% of the ALS patients included in this study did not visit a neurologist at the first symptoms of disease onset. The diagnosis of ALS was prolonged in LO patients visiting an orthopedist. We speculate that this increase in the diagnostic interval in LO patients visiting an orthopedist was due to a lack of bulbar symptoms in the early stages of this disease.

## Background

Amyotrophic lateral sclerosis (ALS) is a progressive neurodegenerative disorder primarily involving motor neurons in the cerebral cortex, brainstem, and spinal cord. The variability in clinical findings early in the course of ALS and the lack of any diagnostic biological marker make absolute diagnosis difficult. The El Escorial criteria (EE) for diagnosing ALS [1] have been widely accepted and were revised (REE) in 2000 to increase their sensitivity [2]. On the therapeutic front, riluzole has been shown to increase survival in controlled trials [3], although the effect is minimal. Percutaneous gastrostomy (PEG) and noninvasive ventilation (NIV) have also been shown to prolong survival and improve the quality of life [4-6]. However, this improvement is still controversial due to questions about the natural history of ALS. In a recent epidemiologic study, recent ALS patients had significantly prolonged survival and slower disease progression compared to patients from older studies [7]. Moreover, a significant increase in survival was shown in 793 Italian patients with ALS observed over a 28-year period [8]. In contrast, no significant change in survival was shown in patients treated more recently, despite the introduction of supportive measures (NIV, PEG, and riluzole) [9]. These studies have investigated the survival of ALS patients; however, little is known about the relationship between the time the patient first seeks medical attention for the initial symptoms of the disease and its influence on the time to actual diagnosis of ALS [10,11]. We conducted a retrospective hospital-based study on the basis of the time it took to attain a final diagnosis of ALS depending on the specialty of the first physician each patient saw and the type of onset symptoms (bulbar onset [BO] and limb [LO] onset).

## Methods

**Figure 1:**
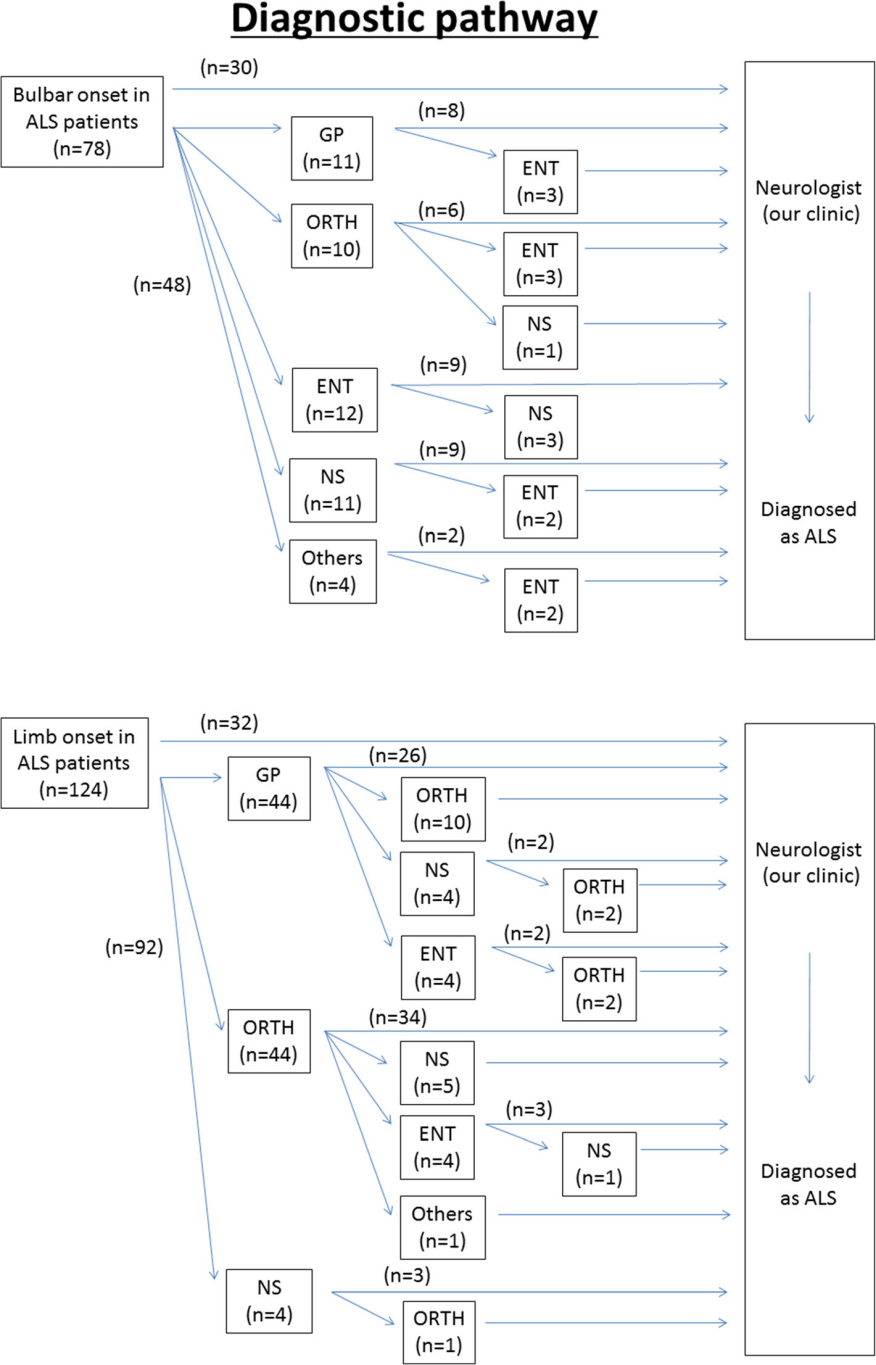
**Diagnostic pathway from symptom onset to final diagnosis of ALS.** GP: general practitioner, ORTH: orthopedist, ENT: otolaryngologist, NS: neurosurgeon.

We reviewed the hospital records of all ALS patients who fulfilled the REE (definite, probable, or probable-laboratory-supported) [2] at Toho University Omori Medical Center, Tokyo, Japan from January 1, 1990 to July 31, 2011. The diagnosis of ALS was made by consulting neurologists with extensive experience (OK, KI, and YI). The patients were stratified according to onset symptoms of ALS, either BO or LO. The type of physician first visited was noted, and the diagnostic interval was calculated as the time between onset of symptoms and the final diagnosis of ALS (Figure 1). Statistical analysis was carried out with the Mann–Whitney *U* test, and P < 0.01 was considered statistically significant. The present study was approved by the Ethical Committee of Toho University Omori Medical Center (Reference no. 23–10).

## Results

### Demographics

A total of 202 patients fulfilled the REE. BO and LO onset symptoms were observed in 78 (36.6%) and 124 (61.4%) patients, respectively. The mean age of symptom onset was 60.8 years (standard deviation [SD] = 11.3; range 26–83); the male to female ratio for all symptoms was 1.6:1, with a breakdown of 1.1:1 in the BO group and 2.1:1 in the LO group. These results are summarized in Table 1.

**Table 1:** Demographics, diagnostic interval, and diagnostic pathway of ALS patients

	**Total**	**Bulbar onset**	**Limb onset**
**Demographics**			
No. of patients (%)	202	78 (36.6)	124 (61.4)
Gender ratio F:M	1:1.6	1:1.1	1:2.1
Mean age of onset in years (SD)	60.8 (11.3)	58.1 (11.0)	63.2 (10.6)
**Diagnostic Interval**			
Average number of months from symptom onset (SD)	13.1 (6.5)	9.2 (4.5)	15.2 (7.7)
**Diagnostic Pathway**			
Neurologist, n (%)	62 (30.7)	30 (38.5)	32 (25.8)
General Practitioner, n (%)	55 (27.2)	11 (14.1)	44 (35.5)
Orthopedist, n (%)	54 (26.7)	10 (12.8)	44 (35.5)
Otolaryngologist, n (%)	12 (5.9)	12 (15.4)	0
Neurosurgeon, n (%)	15 (7.4)	11 (14.1)	4 (3.2)
Others, n (%)	4 (2.0)	4 (5.1)	0

### Diagnostic interval and initial screening physician

The diagnostic interval (SD in parentheses) from symptom onset to diagnosis was 13.1 (6.5) months irrespective of the particular symptoms. The symptom-dependent diagnostic interval was 9.2 (4.5) months in the BO group and 15.2 (7.7) months in the LO group. The patient ratio (BO:LO) was 38.5%:25.8% for patients who visited a neurologist at the first symptoms of disease, 14.1%:35.5% for those who visited a general practitioner (GP), 12.8%:35.5% for those who visited an orthopedist (ORTH), 15.4%:0% for those who visited an otolaryngologist (ENT), 14.1%:3.2% for those who visited a neurosurgeon (NS), and 5.1%:0% for those who visited all other practitioners. More than 50% of the ALS patients examined in this study did not visit a neurologist at the first symptoms of disease (Table 1).

The diagnostic pathway from the onset of symptoms to final diagnosis of ALS is summarized in Figure 1. Fourteen ALS patients visited 2 clinics before finally visiting a neurologist in the BO group. On the other hand, 24 patients visited 2 clinics and 5 patients visited 3 clinics in the LO group.

### Diagnostic interval for patients with BO and LO

**Figure 2:**
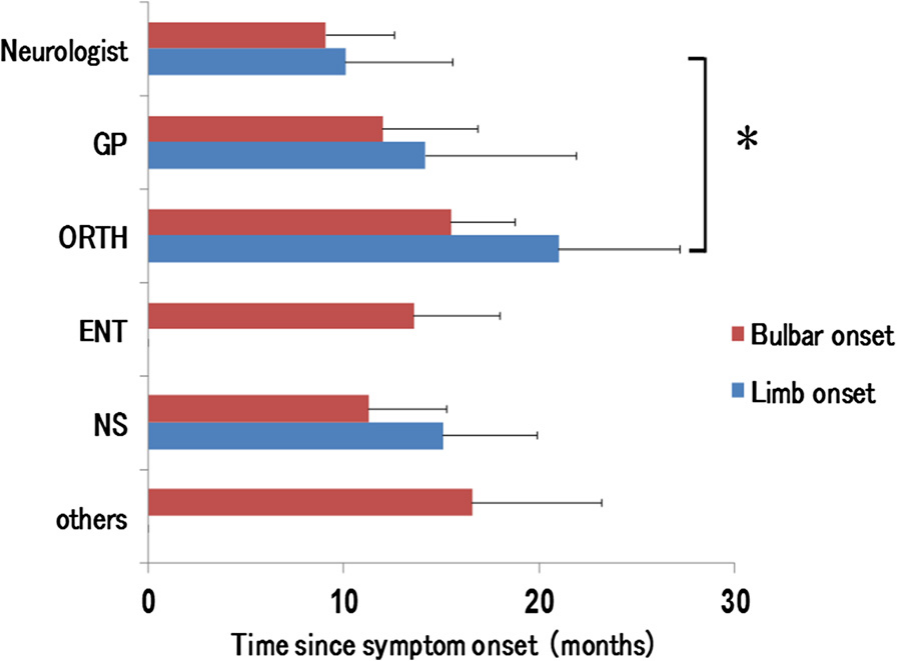
**Diagnostic interval of ALS patients.** The length of time taken for a final diagnosis of ALS from symptom onset. LO patients visiting an ORTH showed a more than 10-month delay in ALS diagnosis compared to those who visited a neurologist after presentation of initial symptoms. *P < 0.01 vs. Neurologist, LO.

The diagnostic interval from symptom onset to final diagnosis is summarized in Figure 2. There were no statistical differences among BO patients. However, the diagnosis of LO was delayed by more than 10 months in patients who saw an ORTH first as compared to those whose initial visit was with a neurologist (P < 0.01). There were no differences between patients who visited a neurologist and those who visited any of the other physicians investigated.

### Direct and indirect referral from an ORTH to a neurologist in LO patients

We analyzed the characteristic differences between patients who were referred to a neurologist directly from an ORTH and those who were referred from an ORTH to other clinics before finally visiting a neurologist (Table 2). Three patients who were referred directly to a neurologist received spinal surgery. However, there were no differences in mean age, spinal spondylosis, or diagnostic interval between these 2 groups.

**Table 2:** Direct and indirect referral from an ORTH to a neurologist in LO patients

	**ORTH to Neurologist**	**ORTH to other clinics to Neurologist**
**Demographics**		
No. of patients (%)	34 (77.3)	10 (22.7)
Gender ratio F:M	1:2.4	1:1.5
Mean age of onset in years (SD)	63.1 (8.2)	61.9 (7.8)
Spinal spondylosis, n (%)	20 (58.9)	5 (50.0)
Spinal surgery, n (%)*	3 (8.8)	0
**Diagnostic Interval**		
Average number of months from symptom onset (SD)	21.9 (6.1)	17.1 (5.5)

*Diagnosed by a neuroradiologist.

## Discussion

The medical system for patients varies from country to country. In Japan, it is recommended that patients have their own primary care doctor; however, they can visit any clinic first freely and without a referral. Once they visit a clinic, the physician makes a referral to the specialist if needed. Therefore, it is difficult to make a country-to-country comparison of the duration between onset of symptoms and diagnosis of ALS.

The diagnosis of ALS is achieved by examination and a series of investigations designed to exclude other clinical syndromes. One of the most valuable aspects to an early ALS diagnosis is the ability to provide the best quality of life for the patient and their caregivers. In addition, it is likely that potential treatment options in the future will have greater effects if administered at the first symptoms of the disease.

Several studies investigating survival [7-9,12-16], diagnostic interval [14], and initial physician contact [10,11] in ALS have been reported. We evaluated only the diagnostic interval in order to exclude the influence of supportive measures and focus on the time lapse that can be induced when diagnoses are made by physicians of various backgrounds. The demographic data of the population of this study was similar to that in previous reports with respect to gender, site of initial symptoms, and the age of onset [13]. We also found that ENT specialists tend to see more BO patients, and ORTH specialists tend to see more LO patients. These findings have also been observed in different countries [10,11]. Diagnostic interval tends to be longer in LO patients compared to BO patients. Our results show that LO patients who visit an ORTH first have a longer diagnostic interval compared to those who visit a neurologist first. Furthermore, no difference could be seen between LO patients who visited a neurologist first and those who visited any of the other physicians investigated. We also compared the characteristic differences between LO patients who were referred to a neurologist directly and those who were referred from an ORTH to other clinics before finally visiting a neurologist. Three patients directly referred to a neurologist received spinal surgery, but there were no differences between these 2 groups in mean age, spinal spondylosis, or diagnostic interval. A high incidence of spondylosis is reported in patients at the mean age of ALS onset, and about 4% of ALS patients undergo decompressive spinal surgery after the onset of retrospectively recognized symptoms of ALS [17]. Therefore, the possibility of ALS must be recognized in the evaluation of weakness, even in the presence of radiographic evidence of spinal spondylosis.

Turner et al. [11] reported the diagnostic pathway and prognosis in BO patients. About 50% of BO patients visited other specialists before visiting a neurologist, but this did not influence diagnostic latency or overall survival. To our knowledge, our study is the first report to describe the relationship between diagnostic pathway, the specialty of the physician initially assessing the ALS patient, and the diagnostic interval in LO patients. We speculate that the increase in diagnostic interval observed in LO patients visiting an ORTH was because of a lack of bulbar symptoms in the early stages of this disease. In addition, ORTH specialists may pay attention to spondylotic myelopathy and radiculopathy, which often coexist in older persons.

## Conclusion

In this study, we have shown that more than 50% of patients with ALS were referred to an inappropriate clinic prior to final diagnosis. When investigating LO ALS patients, those who chose to consult a neurologist first after the onset of symptoms were diagnosed more than 10 months ahead of LO patients who chose to consult an ORTH first. Therefore, we suggest a greater understanding and awareness of ALS through the increased education of non-neurological physicians.

## Abbreviations

ALS: Amyotrophic lateral sclerosis; BO: Bulbar onset; LO: Limb onset; EE: El Escorial criteria; REE: Revised El Escorial criteria; PEG: Percutaneous gastrostomy; NIV: Noninvasive ventilation; GP: General practitioner; ORTH: Orthopedist; ENT: Otolaryngologist; NS: Neurosurgeon.

## Competing interests

The authors declare that they have no competing interests.

## Authors’ contributions

OK, KI, HI, and YK conceptualized the study. DC, KI, and YI participated in coordination and helped draft the manuscript. All authors have read and approved the final manuscript.

## Pre-publication history

The pre-publication history for this paper can be accessed here:

http://www.biomedcentral.com/1471-2377/13/19/prepub
